# Physical Activity among Rural Residents in Eastern, Central, and Western Provinces of China: A Cross-Sectional Survey

**DOI:** 10.1155/2023/7745815

**Published:** 2023-01-23

**Authors:** Cheng-Yao Liang, Zhi-Yuan Zheng, Yu-Gao Wu, Zhuo-Yang Li, Ping Wang, Yi-Yang Wang, Bai-Xue Lin, Jing Fang

**Affiliations:** ^1^Institute for Health Sciences, Kunming Medical University, Kunming 650500, China; ^2^Quality Control Office of Weifang People's Hospital, Weifang 261000, China; ^3^Medical Department of Heze Municipal Hospital, Heze 274000, China

## Abstract

Physical activity (PA) in which physical exercise (PE) is an important component is probably the most important intervention for preventing noncommunicable diseases (NCDs). However, few studies on PA and PE of rural residents in China were reported. This study conducted the first population-based cross-sectional survey in three provinces of China in 2021 that examined both PA and PE as well as the associated factors of rural residents. The International Physical Activity Questionnaire Short Form (IPAQ-S) was used, and a total of 3780 rural residents were surveyed. The result showed that 22.2% of the rural residents were physical inactivity and rural residents reporting practice of PE was 54.4%. Binary logistic regression analyses showed that being female, people aged between 15 to 34 years or 60 years old and above, employees of governmental departments/retirees, school students, the unemployed, and people with NCDs were risk factors of PA while ethnic minority groups, smoking, and alcohol consumption were risk factors of PE. Health promotion programme aiming at increasing people's PA in rural China is urgently needed, and it should focus on the population groups of the female, people aged 60 years and above, school students, the unemployed, and people with NCDs.

## 1. Background

Physical activity (PA) is undoubtedly very important as it has a variety of beneficial effects on physical, mental, and spiritual health and wellness. The World Health Organization (WHO) defines PA as “any bodily movement produced by skeletal muscles which results in energy expenditure above resting level” [1, 2]. The evidence about the health benefits of regular PA is well established [3]. Benefits of PA have been reported for numerous outcomes such as mortality decline [4, 5], cognitive and physical improvement [5–7], glycaemic control [8, 9], relief of pain and disability [10–12], muscle and bone strength [13], reducing depressive symptoms [14], and improving functional mobility and well-being [15, 16]. Physical exercise (PE) is an important source of PA. The benefits of PE to human body include but not limited to immunological [17], musculoskeletal [18], respiratory [19], and hormonal aspects [20]. Specifically for the cardiovascular system, PE increases fatty acid oxidation, cardiac output, vascular smooth muscle relaxation, endothelial nitric oxide synthase expression, and nitric oxide availability and improves plasma lipid profiles [16] while at the same time reducing resting heart rate and blood pressure, aortic valve calcification, and vascular resistance [21].

Noncommunicable diseases (NCDs), also known as chronic conditions or chronic diseases, are long lasting diseases. The main types of NCDs include cardiovascular diseases, cancers, musculoskeletal diseases, chronic respiratory diseases, mental illness, and diabetes. The ever-growing prevalence of NCDs has become a global health concern [22]. According to a report published by the World Health Organization (WHO) in 2019, the top 10 causes of death accounted for 55% of the 55.4 million deaths worldwide and 7 of the 10 leading causes of deaths were NCDs [23]. In China, there was 9.05 million NCDs deaths in 2019 [24], and about 82% of China's disease burden is due to the prevalence of NCDs [25]. Global Health Observatory Data issued by the WHO has listed risk factors for NCDs including physical inactivity, alcohol intake, tobacco use, hypertension, obesity, raised cholesterol, unhealthy diet, and raised blood glucose [26]. Of these factors, physical inactivity is considered as the fourth leading risk factor for global mortality [27]. In 2015, physical inactivity directly contributed to 21% of breast cancers, 25% of colon cancers, 27% of diabetes, and 30% of ischemic heart diseases globally [28]. Recent estimates indicate that 9% of the overall global premature mortality, ≈5.3 million deaths, is directly attributable to physical inactivity, a figure comparable to the global smoking-related mortality (5.1 million) [29]. Conservatively estimated, physical inactivity cost health-care systems $53.8 billion worldwide in 2013, of which $31.2 billion was paid by the public sector, $12.9 billion by the private sector, and $9.7 billion by households. In addition, physical inactivity-related deaths contribute to $13.7 billion in productivity losses, and physical inactivity was responsible for 13.4 million disability-adjusted life years (DALYs) worldwide [30].

Despite the well-acknowledged benefits linked to PA, a significant proportion of the global population remains physically inactive. Globally, in 2016 among the 1.9 million individuals providing self-reported PA levels via the International Physical Activity Questionnaire (IPAQ), the global prevalence rate of physical inactivity was 27.5%, which was slightly smaller than 28.5%, the prevalence rate of physical inactivity in the globe in 2001 [31], showing no progress has been made in PA of global population during a period of 15 years. However, the prevalence rates of physical inactivity vary across the groups of low-income, middle-income, and high-income countries, with 15.5%, 27.5%, and 31.1% of adult populations reporting physical inactivity, respectively [32]. Furthermore, in a few high-income countries, low levels of adult participation in PA are much more concerning [3]. A study reported that globally, older adults (aged 60 years and above) are the least active population group: the prevalence rate of physical inactivity of this group was 55%, and only 45% of them met the WHO recommendation for 150 weekly minutes of moderate-to-vigorous physical activity for achieving health benefits [33].

Reducing physical inactivity is a challenge for many nations. As a rising developing country with a big population, China faces the challenge of climbing NCDs in which physical inactivity may be a significant contributor. One national survey reported that Chinese citizens aged 7 years and above practicing regular PE merely 37.2% in 2020 [34] while another survey of urban residents conducted in Liaoning Province of China in 2018 reported that the prevalence rate of physical inactivity was 25.3% [35]. Although the two studies focused on different issues (PE versus PA), both studies indicate the possible lower level of PA among Chinese. Until now, there has no nationwide survey on people's PA in China been reported although several studies on PA in certain areas of China could be found and majority of those studies focused on urban dwellers of economically more developed regions [36–38]. What is the PA status of rural residents in China? What percentage of rural residents is physically inactive? What percentage of rural residents practices regular PE? What are the associated factors? It is very imperative and important to explore answers to these questions in order to better prevent and control NCDs in rural China. Therefore, this study used the International Physical Activity Questionnaire Short Form (IPAQ-S) [39] to conduct a questionnaire survey in rural areas of Shandong, Shanxi, and Yunnan provinces representing eastern, central, and western region of China in order to understand the current status of PA and its influencing factors as well as frequency and pattern of PE among rural residents in China. The findings of this study will reflect the overall status of PA, PE, and physical inactivity of the rural residents in China, which will fill out the current research gap and more important be helpful for the design of interventions to reduce physical inactivity among Chinese rural residents.

## 2. Methods

### 2.1. Study Design

**Figure 1:**
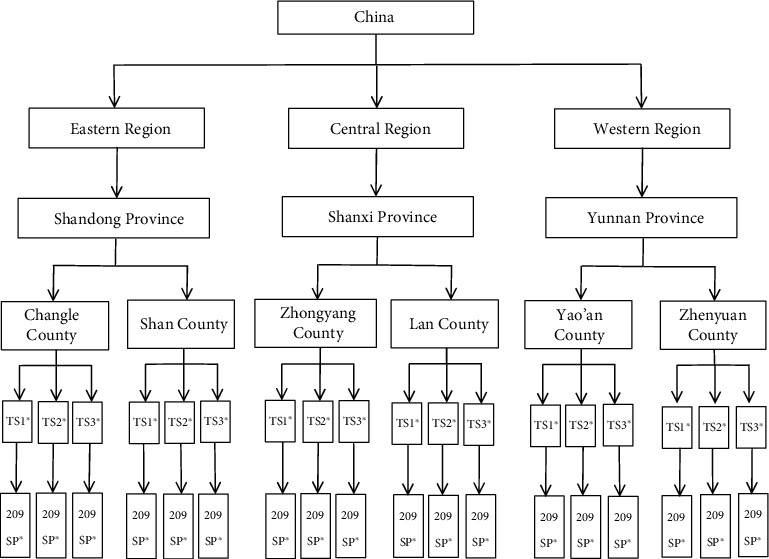
The sampling process from province to survey participants. ^∗^TS: township; SP: survey participants.

We planned and conducted a population-based cross-sectional survey and used a multistage random sampling method. We first selected one province, respectively, from the eastern, central, and western regions of China and secondly selected two counties from each of the three selected provinces, which produced a sample of six counties located in three provinces. Finally, Changle County of Weifang City and Shan County of Heze City in Shandong Province, Zhongyang County and Lan County of Lvliang City in Shanxi Province, Yao'an County of Chuxiong Yi Autonomous Prefecture, and Zhen Yuan Yi Hani Lahu Autonomous County of Pu'er City in Yunnan Province were selected as sampling counties for the study. The multistage random sampling method was employed in each sampled county to get the sample of rural residents: three townships were randomly selected in each county where rural residents aged above 15 years old living there for more than 6 months were randomly selected to participate in the survey.  Figure 1 illustrates the sampling process from province to survey participants at township level. All participants were informed the purpose and content of this survey, and informed consent was obtained from each participant. The survey was conducted in a face-to-face manner, and the investigators were postgraduate students and teachers from the School of Public Health at Kunming Medical University who had been well trained prior to the survey. The survey was approved by the Research Ethic Review Committee of Kunming Medical University.

For the assessment of self-reported PA, the IPAQ was employed by many studies. IPAQ is an instrument designed to assess levels of PA, and short and long forms of the questionnaire have been developed on the basis of self-report population surveys [40], and the instrument has been validated in different languages [41–43]. In the present study, the IPAQ-S was used. This questionnaire comprises of seven questions that assess the frequency and duration of vigorously intense physical activity (VPA), moderately intense physical activity (MPA), and walking for at least 10 min during the past week. The IPAQ-S assesses total weekly PA, whereas the intensity of activity is converted to metabolic equivalent of task (MET) units, as recommended by previous study [44]. Guidelines for data processing and analysis of the International Physical Activity Questionnaire (IPAQ) describe recommended methods of scoring the data derived from the IPAQ-S. According to the guidelines for data processing and analysis of the International Physical Activity Questionnaire (IPAQ), the selected MET values were derived from work undertaken during the IPAQ reliability study. MET score was derived for each type of activity. For example, all types of walking were included, and MET value for walking was created. The same procedure was undertaken for MPA and VPA. The following values continue to be used for the analysis of IPAQ data: walking = 3.3 METs, MPA = 4.0 METs, and VPA = 8.0 METs. Using these values, four continuous scores are defined: (1) walking MET × min/wk = 3.3 × walking minutes/day × walking days/week; (2) MPA MET × min/wk = 4.0 × MPA minutes/day × moderate − intensity days/week; (3) VPA MET × min/wk = 8.0 × VPA minutes/day × vigorous − intensity days/week; and (4) total PA MET × min/wk = sum of (walking + MPA + VPA MET) × minutes/week scores [40]. In this study, the English version of the IPAQ-S was translated into Chinese and adapted to the local situation in rural China to form the PA questionnaire for Chinese rural residents. The questionnaire also includes questions about the pattern, length, and frequency of PE among rural residents.

### 2.2. Sample Size of Rural Residents

In order to estimate the sample size of rural residents, we used the equation: *n* = ((*μ*_(1 − (*α*/2))_^2^ × *p*(1 − *p*))/*δ*^2^) × deff, in which *p* = 37.2% that was the proportion of people aged 7 years and above who practiced PE regularly reported by a national survey conducted in China in 2020 [34], *δ* = *p* × 20% = 0.0744, *α* = 0.05, *μ* (1 − *α*/2) = 1.96, and design efficiency deff = 1.5; then, the estimated sample size *n* was 199 survey participants per township. Considering the possible invalid questionnaires, we expanded the actual sample size by 5%, and finally, we obtained the sample size of 209 survey participants in each township and 627 survey participants in each county.

### 2.3. Variables and Outcomes Measurements

Data on gender, ethnicity, age, marital status, educational attainment, occupation, annual per capital household income, status of NCDs, and lifestyle factors, such as smoking and alcohol consumption, were collected. Ethnicity was categorised into two groups: the Han group and ethnic minority group. Age was categorised into three groups: 15-34 years (young group), 35-59 years (middle-aged adults), and 60 years and above (old adults). Marital status was categorised into four groups: single, married, widowed, and divorced. Educational attainment was divided into five groups following the school system in China: illiterate or have little literacy, primary school, junior high school, high school, and undergraduate and above. Occupation was divided into five groups: farmers/migrant workers, business men/service workers, employees of governmental departments/retirees, school students, and unemployed. Annual per capital household income was divided into 4 groups: less than 5000 Chinese yuan (CNY), equal to or greater than 5000 but less than 10000 CNY, equal to or greater than 10000 but less than 20000 CNY, and 20000 CNY and above. Criteria for NCDs are as follows: reported by survey participants that was supported with diagnosis from hospitals at county and above level. MET is the common unit used internationally to reflect the absolute intensity of PA [45]. A MET is defined as the amount of consumed oxygen at rest by a person, which is approximately 3.5 ml O_2_/kg/min. Calculation of total PA was done according to the guidelines for data processing and analysis of the IPAQ-S [40]: a MET value of 3.3 was assigned for walking, a MET value of 4.0 was assigned for moderately intense physical activity (MPA), and a MET value of 8.0 was assigned for vigorously intense physical activity (VPA). Four equations [40, 46] of PA in the guidelines for data processing and analysis of the IPAQ-S were defined: (1) walking MET × min/wk = 3.3 × walking minutes/day × walking days/week; (2) MPA MET × min/wk = 4.0 × MPA minutes/day × moderate − intensity days/week; (3) VPA MET × min/wk = 8.0 × VPA minutes/day × vigorous − intensity days/week; and (4) total PA MET × min/wk = sum of (walking + MPA + VPA MET) × minutes/week scores. According to the WHO guidelines [47], physical inactivity was defined as not meeting any of the following three criteria: (1) 30 min of moderate-intensity PA on at least 5 days every week; (2) 20 min of vigorous-intensity PA on at least 3 days every week; and (3) an equivalent combination achieving 600 MET per week.

### 2.4. Statistical Analysis

The descriptive data of the survey participants was expressed as frequencies and percentages. The chi-squared test was used to compare frequencies and percentages between groups. Lastly, binary logistic regression was used to explore the influencing factors of PA and PE. The main outcomes of interest were the dichotomous dependent variables of PA (1 = physically inactive and 0 = physically active) and PE (1 = no and 0 = yes). The following variables were included into the binary logistic regression model: (1) gender, (2) ethnicity, (3) age, (4) marital status, (5) educational attainment, (6) occupation, (7) annual per capital household income, (8) smoking, (9) alcohol consumption, and (10) status of NCDs. All statistical analyses were performed using IBM SPSS for Statistics (version 24; SPSS Inc. (version 24.0 Armonk, NY, USA)). For all statistical analyses, *p* ≤ 0.05 was considered statistically significant.

## 3. Results

### 3.1. Demographic Characteristics of Survey Participants

A total of 3780 rural residents (response rate 98.6%) participated in the survey. Their median (IQR) age was 53 (P25: 39 and P75:65) years with 46.9% aged between 35 and 59 years and 35.0% aged 60 years and above. Of the total survey participants, 49.4% were male and 50.6% were female. The Han ethnic group accounted for 90.1%. Regarding educational status, 49.8% were below secondary school and very few (1.5%) had undergraduate and above level. Majority of the survey participants (86.5%) were married. In terms of occupation, 56.3% were farmers/migrant workers and 24.6% were the unemployed. The median (IQR) annual per capita household income was 9000.00 (P25:3333.33 and P75:17142.85) CNY. Besides, there were 1340 (35.4%) survey participants with NCDs in this study. Details about demographic characteristics of the survey participants are shown in Table 1.

### 3.2. PA of Survey Participants

The result showed that although 77.8% of the all survey participants were physically active, 22.2% were physical inactivity, of which the female had higher prevalence rate of physical inactivity (25.2%) than their male counterparts (19.1%) (*χ*^2^ = 20.520, *p* < 0.001). The prevalence rate of physical inactivity of the Han ethnic group (23.3%) was higher than that of ethnic minority groups (12.3%) (*χ*^2^ = 23.418, *p* < 0.001). In terms of occupation, the prevalence rate of physical inactivity was highest among the unemployed, at 37.4%, and that prevalence rate of physical inactivity of employees of governmental departments/retirees was 24.5% (*χ*^2^ = 187.695, *p* < 0.001). Among age groups, old adults (aged 60 years and above) were the most inactive group of the population (27.8%) (*χ*^2^ = 51.415, *p* < 0.001), compared to young group and middle-aged adults. The prevalence rate of physical inactivity of the survey participants with NCDs was 27.8%, higher than that of survey participants without NCDs (19.1%) (*χ*^2^ = 37.848, *p* < 0.001). The different prevalence rates of physical inactivity among different annual per capita household income groups, smoking groups, and alcohol consumption groups were also statistically significant by chi-squared test. Details about PA of the survey participants by demographic variables are shown in Table 2.

### 3.3. Influencing Factors of PA of Survey Participants

We entered the variables presented in Table 2 that were statistically significant by chi-squared test into the model of binary logistic regression analysis, and the results showed that gender, ethnicity, age, occupation, and status of NCDs were the influencing factors of PA. As shown in Table 3, ethnic minority groups (OR = 0.559, 95% CI, 0.401-0.778) was protective factor of PA while female (OR = 1.307, 95% CI, 1.107-1.543), people aged between 15 and 34 years (OR = 1.426, 95% CI, 1.119-1.818) or 60 years and above (OR = 1.351, 95% CI, 1.113-1.641), employees of governmental departments/retirees (OR = 1.603, 95% CI, 1.127-2.282), school students (OR = 2.141, 95% CI, 1.255-3.650), the unemployed (OR = 2.461, 95% CI, 2.051-2.954), and people with NCDs (OR = 1.430, 95% CI, 1.188-1.721) were risk factors of PA.

### 3.4. PE of Survey Participants

In this survey, the proportion of rural residents who reporting practice of PE was 54.4%. Among those reporting practice of PE, 1582 survey participants (76.8%) practiced PE five times and above per week and 479 survey participants (23.2%) practiced PE less than five times per week. About 93.8% of survey participants practiced PE for 30 minutes and above each time while the rest 6.2% practiced PE for less than 30 minutes. The most frequently performed PE was walking/brisk walking (1614 participants, 78.3%). There were 389 (18.9%), 336 (16.3%), 104 (5.0%), and 65 (3.2%) of the survey participants reporting practice of running, dancing/calisthenics, mountain climbing, and badminton/table tennis, respectively, while fitness and t'ai chi ch'uan were the least reported PE practiced only by 20 (1.0%) and 11 (0.6%) participants, respectively. Details about PE of the survey participants are shown in Table 4.

### 3.5. Influencing Factors of PE of Survey Residents

We entered the same variables presented in Table 2 that were statistically significant by chi-squared test into the model of binary logistic regression analysis of PE, and the results showed that ethnicity, age, occupation, educational attainment, smoking, and alcohol consumption were the influencing factors of PE. As shown in Table 5, people aged between 35 and 59 years (OR = 0.590, 95% CI, 0.478-0.728) or 60 years and above (OR = 0.416, 95% CI, 0.329-0.528), employees of governmental departments/retiree (OR = 0.359, 95% CI, 0.249-0.519), school students (OR = 0.086, 95% CI, 0.038-0.195), the unemployed (OR = 0.490, 95% CI, 0.414-0.580), and people with education level of high school (OR = 0.603, 95% CI, 0.464-0.784) or undergraduate and above (OR = 0.346, 95% CI, 0.162-0.739) were protective factors of PE while ethnic minority groups (OR = 1.525, 95% CI, 1.210-1.921), smoking (OR = 1.173, 95% CI, 1.000-1.375), and alcohol consumption (OR = 1.371, 95% CI, 1.151-1.633) were risk factors of PE.

## 4. Discussion

### 4.1. Status of PA among Rural Residents of China

PA is probably the single most important intervention for preventing NCDs, making it one of the primary determinants of health [48]. The WHO recommends at least 150 min of MPA per week, i.e., 30 min a day, 5 days a week, for all adults [49]. Our survey showed that 22.2% of the rural residents in three provinces of China were physical inactivity, which was slightly smaller than the level of physical inactivity in Liaoning Province of China in 2018, reported as 25.3% [35]. This indicates that nearly a quarter of rural residents in three provinces of China failed to meet the recommended PA level to achieve health benefits, leading to negative effects on the general health and rising NCDs risk of rural residents in China.

### 4.2. Gender Is an Influencing Factor of PA

This study examined a number of factors such as gender, ethnicity, age, occupation, and status of NCDs that may have impact on the PA of rural residents in China. Among others, gender had significant impact on PA. Our survey found that female rural residents were less physically active than their male counterparts. The prevalence rate of physical inactivity of the female was 25.2%, higher than the male (19.1%). Previous studies reported similar findings [50–52]. This might be caused by traditional customs and gender roles. In most rural households, women are typically assumed the role of cooking, cleaning, and caring for children and the elderly in the family [51]; those tasks are typically less physically intensive and thus may not be well captured by the IPAQ-S. Compared with women, men usually more engage in heavier agricultural activities, related to crop growing and harvest such as carrying chemical fertilizers to field.

### 4.3. Age Is an Influencing Factor of PA

Our results showed that age had significant impact on PA. Among the survey participants, the prevalence rate of physical inactivity of old adults (aged 60 years and above) was the highest (27.8%) while only 17.2% of middle-aged adults (aged 35-59 years) were physical inactivity. Many studies reported age-related decline in PA [50, 53, 54]. Because of ageing, old adults tend to face more constraints on participation in PA than their younger peers [55]. The biological process of aging is likely to be the reason for this outcome. Old adults tend to face a more serious deterioration in health and thus have greater difficulties in performing PA. They are more likely to perform less intensity PA, which contribute to low level of PA of old adults.

### 4.4. Occupation Is an Influencing Factor of PA

In the present survey, occupation had impact on PA. The finding was similar with previous studies [56–58]. We found out that the unemployed and school students had lower level of PA. One plausible explanation could be that the unemployed have fewer work commitments and often live a laid-back lifestyle and consequently would be less physically active. As for the student group, they face strong cultural and social pressure to achieve academic excellence and thus have little time to spend on physical activities. This result is in line with previous study that consistently showed disappointingly low levels of PA among Chinese students [59], and being physically underactive can increase the likelihood of developing unhealthy of school students in China.

### 4.5. NCDs Have Impact on the PA of Rural Residents

The results of the present survey showed that the prevalence rate of physical inactivity of rural residents with NCDs was 27.8%, higher than those without NCDs (19.1%). Similar findings were reported by previous studies. NCDs, such as cardiovascular and neurological diseases, were events that are strongly associated with physical inactivity, which produced catabolic effects on the muscles and nervous and cardiovascular systems, which in conjunction favor the physical performance decline [60–63]. Besides, decreased mobility from joint diseases was a limiting factor for adherence to PA in rural residents with NCDs [64]. The likelihood of being insufficiently active was higher in those having NCDs [63, 65].

### 4.6. Ethnicity Is an Influencing Factor of PA

We found that ethnicity had a significant effect on PA in China. The prevalence rate of physical inactivity of Han ethnic group (23.3%) was significantly higher than the one of ethnic minority groups (12.3%). One plausible reason was that the majority of surveyed Han ethnic group live in the more economically developed eastern and central regions of China where most agricultural activities are undertaken with machine while most of surveyed ethnic minority groups reside in the less developed western region of China where most agricultural activities are still manually conducted. The level of individuals' income of Han ethnic group was higher than that of ethnic minority groups. Previous studies [66–68] showed that the higher the level of individuals' income, the less likely that individuals being physically active. Most of higher income individuals are usually engaged with jobs that are at low-intensity PA that cause lower level of PA.

### 4.7. PE of Rural Residents and Associated Factors

PE is an important source of PA [69]. We found 54.4% of survey participants reporting practice of PE at least once a week, and the most commonly performed PE was walking/brisk walking (78.3%). We also found that unhealthy lifestyle habits, such as smoking and alcohol consumption, were risk factors of PE. According to General Administration of Sport of China, in 2020, 63.1% of rural residents in China practiced PE at least once a week and walking/brisk walking (22.7%) and running (19.8%) were the top two commonly practiced PE by adults in China that was followed by badminton (8.9%), cycling (7.3%), and basketball (5.4%) [70]. The proportion of rural residents practicing PE in our survey is smaller than 63.1%. Interestingly, the logistic regression analysis result showed that those groups who were less physically active such as the unemployed, employees of governmental departments/retirees, students, and the elderly are more likely to undertake PE, which may be a way to compensate their physical inactivity and also a potential entry point for intervention. However, the current ways of PE adopted by rural residents were simple with little requirements for equipment and space that may be determined by the less developed sports and PE facilities in rural areas. Due to the lack of sports and recreational infrastructure and inadequate built environments that can facilitate badminton/cycling/basketball and other forms of PE in rural areas of China, rural residents have little choice but can only adopt simple forms of physical exercise such as walking and running and their enthusiasm for PE can be reduced. With further economic development and people shifting away from organically incorporating PA in daily work activities, people would need to pursue PA more proactively through PE [71]. Government should pay more attention to build sport and PE facilities in rural areas that will improve PE level of rural residents.

## 5. Limitations

There are some limitations in this study; therefore, our findings should be interpreted with certain caution. First, this study is limited by its cross-sectional design, and thus, it precludes any conclusions on causality. Second, this study estimated the volume of PA using IPAQ-S that requires survey participants to self-report and recall their PA of the last week, which can produce recall bias, and because the survey was conducted in a face-to-face manner, the bias caused by social desirability could also be produced. Third, although the use of a validated instrument to evaluate PA may be regarded as a strength of this study, some studies have indicated that the IPAQ could overestimate the total PA, when compared with data measured by an objective method [72, 73]. Future studies are needed that should validate our findings by utilizing longitudinal data with repeated measurement of PA preferably based on objective measurements.

## 6. Conclusions

We conducted a survey to examine both PA and PE status as well as the associated factors of rural residents in China. Around a quarter of rural residents (22.2%) were physical inactivity, and the rural residents reporting practice of PE were only 54.4%. The survey revealed that gender, ethnicity, age, occupation, and status of NCDs were the influencing factors of PA of rural residents in China while ethnicity, age, occupation, educational attainment, smoking, and alcohol consumption were the influencing factors of PE of those populations. The results of this survey provide useful information to inform the design and development of policies and interventions for the promotion of PA among rural residents in China. Health promotion programme aiming at increasing people's PA in rural China should focus on the populations of the female, people at age 60 years and above, school students, the unemployed, and people with NCDs.

## Figures and Tables

**Table 1:** Demographic characteristics of survey participants (*n* = 3780).

Variables		*n*	%
Gender	Male	1867	49.4
	Female	1913	50.6

Ethnicity	Han ethnic group	3407	90.1
	Ethnic minority groups	373	9.9

Age (years)	15-34	685	18.1
	35-59	1773	46.9
	60 and above	1322	35.0

Marital status	Single	201	5.3
	Married	3271	86.5
	Widowed	283	7.5
	Divorced	25	0.7

Educational attainment	Illiterate or have little literacy	1006	26.5
	Primary school	879	23.3
	Junior high school	1318	34.9
	High school	521	13.8
	Undergraduate and above	56	1.5

Occupation	Farmers/migrant workers	2127	56.3
	Business men/service workers	452	12.0
	Employees of governmental departments/retirees	192	5.1
	School students	77	2.0
	The unemployed	932	24.6

Annual per capita household income (CNY)	0 ~	1414	37.4
	5000 ~	835	22.1
	10000 ~	862	22.8
	20000 ~	669	17.7

Status of NCDs	Yes	1340	35.4
	No	2440	64.6

**Table 2:** PA status of survey participants (*n* = 3780).

Variables		Physically active		Physically inactive		Chi-squared value	*p* value
		*n*	%	*n*	%		
Total		2940	77.8	840	22.2		

Gender	Male	1510	80.9	357	19.1	20.520	<0.001
	Female	1430	74.8	483	25.2		

Ethnicity	Han ethnic group	2613	76.7	794	23.3	23.418	<0.001
	Ethnic minority groups	327	87.7	46	12.3		

Age (years)	15-34	517	75.5	168	24.5	51.415	<0.001
	35-59	1468	82.8	305	17.2		
	60 and above	955	72.2	367	27.8		

Marital status	Single	145	72.1	56	27.9	22.952	<0.001
	Married	2582	78.9	689	21.1		
	Widowed	192	67.8	91	32.2		
	Divorced	21	84.0	4	16.0		

Educational attainment	Illiterate or have little literacy	744	74.0	262	26.0	12.028	0.017
	Primary school	701	79.7	178	20.3		
	Junior high school	1039	78.8	279	21.2		
	High school	413	79.3	108	20.7		
	Undergraduate and above	43	76.8	13	23.2		

Occupation	Farmers/migrant workers	1765	83.0	362	17.0	187.695	<0.001
	Business men/service workers	395	87.4	57	12.6		
	Employees of governmental departments/retirees	145	75.5	47	24.5		
	School students	52	67.5	25	32.5		
	The unemployed	583	62.6	349	37.4		

Annual per capita household income (CNY)	0 ~	1045	73.9	369	26.1	21.738	<0.001
	5000 ~	670	80.2	165	19.8		
	10000 ~	678	78.7	184	21.3		
	20000 ~	547	81.8	122	18.2		

Smoking	Yes	958	80.4	234	19.6	6.764	0.009
	No	1982	76.6	606	23.4		

Alcohol consumption	Yes	720	83.5	142	16.5	21.352	<0.001
	No	2220	76.1	698	23.9		

Status of NCDs	Yes	967	72.2	373	27.8	37.848	<0.001
	No	1973	80.9	467	19.1		

**Table 3:** The results of binary logistic regression analysis for influencing factors of PA of survey participants.

Variables		*β*	S.E.	*p* value	OR (95% CI)
Gender	Male				Ref
	Female	0.268	0.085	0.002	1.307 (1.107-1.543)

Ethnicity	Han ethnic group				Ref
	Ethnic minority groups	-0.582	0.169	0.001	0.559 (0.401-0.778)

Age (years)	35-59				Ref
	15-34	0.355	0.124	0.004	1.426 (1.119-1.818)
	60 and above	0.301	0.099	0.002	1.351 (1.113-1.641)

Occupation	Farmers/migrant workers				Ref
	Business men/service workers	-0.301	0.158	0.057	0.740 (0.544-1.009)
	Employees of governmental departments/retirees	0.472	0.180	0.009	1.603 (1.127-2.282)
	School students	0.761	0.272	0.005	2.141 (1.255-3.650)
	The unemployed	0.901	0.093	<0.001	2.461 (2.051-2.954)

Status of NCDs	No				Ref
	Yes	0.358	0.094	<0.001	1.430 (1.188-1.721)

**Table 4:** Frequency, duration, and pattern of PE of survey participants.

Ways of physical exercise	%	Frequency of exercise per week	%	Duration of each time	%
Walking/brisk walking	78.3	Less than 5 times	23.2	<30 min	6.2
Running	18.9				
Dancing/calisthenics	16.3	5 times	5.6	30-60 min	73.5
Mountain climbing	5.0				
Badminton/table tennis	3.2	More than 5 times	71.2	>60 min	20.3
Skipping rope/shuttlecock	2.9				
Basketball/football/volleyball	1.9				
Bicycle	1.6				
Fitness	1.0				
T'ai chi ch'uan	0.6				

**Table 5:** The results of binary logistic regression for influencing factors of PE of survey participants.

Variables		*β*	S.E.	*p* value	OR (95% CI)
Ethnicity	Han ethnic group				Ref
	Ethnic minority groups	0.422	0.118	<0.001	1.525 (1.210-1.921)

Age (years)	15-34				Ref
	35-59	-0.527	0.107	<0.001	0.590 (0.478-0.728)
	60 and above	-0.876	0.121	<0.001	0.416 (0.329-0.528)

Occupation	Farmers/migrant workers				Ref
	Business men/service workers	-0.020	0.110	0.852	0.980 (0.790-1.216)
	Employees of governmental departments/retirees	-1.024	0.188	<0.001	0.359 (0.249-0.519)
	School students	-2.449	0.414	<0.001	0.086 (0.038-0.195)
	The unemployed	-0.713	0.086	<0.001	0.490 (0.414-0.580)

Educational attainment	Illiterate or have little literacy				Ref
	Primary school	0.066	0.099	0.508	1.068 (0.879-1.296)
	Junior high school	-0.228	0.096	0.018	0.796 (0.659-0.961)
	High school	-0.506	0.134	<0.001	0.603 (0.464-0.784)
	Undergraduate and above	-1.061	0.387	0.006	0.346 (0.162-0.739)

Smoking	No				Ref
	Yes	0.159	0.081	0.050	1.173 (1.000-1.375)

Alcohol consumption	No				Ref
	Yes	0.316	0.089	<0.001	1.371 (1.151-1.633)

## Data Availability

The data used to support the findings of this study are available from the corresponding author upon request.
